# Novel Low-Cost Sensor for Human Bite Force Measurement

**DOI:** 10.3390/s16081244

**Published:** 2016-08-06

**Authors:** Jarred Fastier-Wooller, Hoang-Phuong Phan, Toan Dinh, Tuan-Khoa Nguyen, Andrew Cameron, Andreas Öchsner, Dzung Viet Dao

**Affiliations:** 1 School of Engineering, Griffith University, Queensland 4215, Australia; jarred.fastier-wooller@griffithuni.edu.au (J.F.-W.); a.oechsner@griffith.edu.au (A.Ö.); 2 Queensland Micro- and Nanotechnology Centre, Griffith University, Queensland 4111, Australia; hoangphuong.phan@griffithuni.edu.au (H.-P.P.); toan.dinh@griffithuni.edu.au (T.D.); khoa.nguyentuan@griffithuni.edu.au (T.-K.N.); 3 School of Dentistry, Griffith University, Queensland 4215, Australia; a.cameron@griffith.edu.au

**Keywords:** bite force, strain gauge, acrylic, oral health, J0101

## Abstract

This paper presents the design and development of a low cost and reliable maximal voluntary bite force sensor which can be manufactured in-house by using an acrylic laser cutting machine. The sensor has been designed for ease of fabrication, assembly, calibration, and safe use. The sensor is capable of use within an hour of commencing production, allowing for rapid prototyping/modifications and practical implementation. The measured data shows a good linear relationship between the applied force and the electrical resistance of the sensor. The output signal has low drift, excellent repeatability, and a large measurable range of 0 to 700 N. A high signal-to-noise response to human bite forces was observed, indicating the high potential of the proposed sensor for human bite force measurement.

## 1. Introduction

The maximum bite force (MBF) and maximal voluntary bite force (MVBF) of the human jaw can correlate to the wellbeing and oral health of the patient [1,2,3,4]. The literature in this field of study indicates a number of research studies being performed on the relation to or measurement of human and animal bite forces [2,5,6,7,8,9]. For example, MBF has been used to verify the connection between the poor dental health of children and the impact it can have on their quality of life [2]. Bite force has also been employed as an indicator for patients with bruxism, where the patient is able to wear a prescription splint to sleep that has an integrated sensor and circuit to record and relay important information on the patient’s jaw activity while sleeping [6]. Furthermore, an oral occlusion measurement system can give dentists the ability to perform a full analysis on the occlusion of their patients upper and lower jaws, where MBF measurements are only a part of the occlusion measurement process [10]. As such, a full occlusive analysis can be used for assisting in dental rehabilitation through implants and or prosthetics [11].

Finite element analysis (FEA) of a patients’ mouth can be performed using a scanned model of the patient’s teeth and jaw, which presents a potential MBF under various assumptions. However, FEA is not quantifiable as a MVBF since it often tests the physical limits of the size and estimated mass of the patient’s teeth [12], and does not take into account other anatomical structures such as periodontal ligaments and the natural flex of the mandible [13]. Moreover, MVBF measurements can be performed locally in the patient’s mouth and requires physical exertion on the sensor involved. Many factors have an impact on the measured MVBF, such as the position of the device when taking measurements, the opening of the mouth, and the effect of unilateral and bilateral biting. In theory, the highest reading would be taken using a bilateral device positioned at the rear molars [14]. Both unilateral and bilateral in vivo tests are commonly performed in many studies.

To date, there have been a large number of sensors developed to measure a patient’s bite force or MVBF. One such example is T-Scan—a commercially available system which uses a pressure sensitive sheet sensor that is capable of assisting dentists perform a full occlusive analysis on their patients. However, the cost of the T-Scan system is relatively expensive, especially when full occlusive analysis is not necessary. This system has been tested and validated in [10], and has been used by dentists in real-world applications. Another method uses FlexiForce, which is a thin and flexible printed circuit used in force measurements from TekScan Inc. [15]. The Force Sensing Resistor from Interlink Electronics is comparable with FlexiForce in both thickness and application methods [16,17]. FlexiForce and Force Sensing Resistors have been used in the very accurate measurement of bite forces in both humans and animals alike [9,18,19,20]. The bite force range generally falls within 0 to 700 N which is suitable for taking MVBF measurements. However, the output of the system incorporates high drift of approx. 3%–12% in relation to the time and force applied [21]. The drift becomes more significant at larger measurement ranges (e.g., more than 300 N) [15], which may result in inconsistent or invalid measurements.

To avoid signal drift, the system in [2] has employed a bite sensing device using a strain gauge connected to two load bearing beams in order to measure the MVBF. However, this system is still very bulky in both its size and mass, and its fabrication has involved complex steps, thereby constraining its utilization for specific on-site applications.

This paper presents the design, fabrication, and characterization of a novel bite force sensor based on the application of strain gauges. The proposed sensor possesses a compact structure, which can be easily fabricated using a simple fabrication process and a low cost and bio-compatible material. The proposed sensor is capable of measuring a large bite force range of up to 700 N with high repeatability, low drift, and good linearity. We have also successfully demonstrated the use of the sensor for accurate real-time measurement of human bite forces with high signal-to-noise ratio. These results indicate our proposed sensors’ significance for ubiquitous bite force sensing applications.

## 2. Design and Simulation

**Figure 1:**
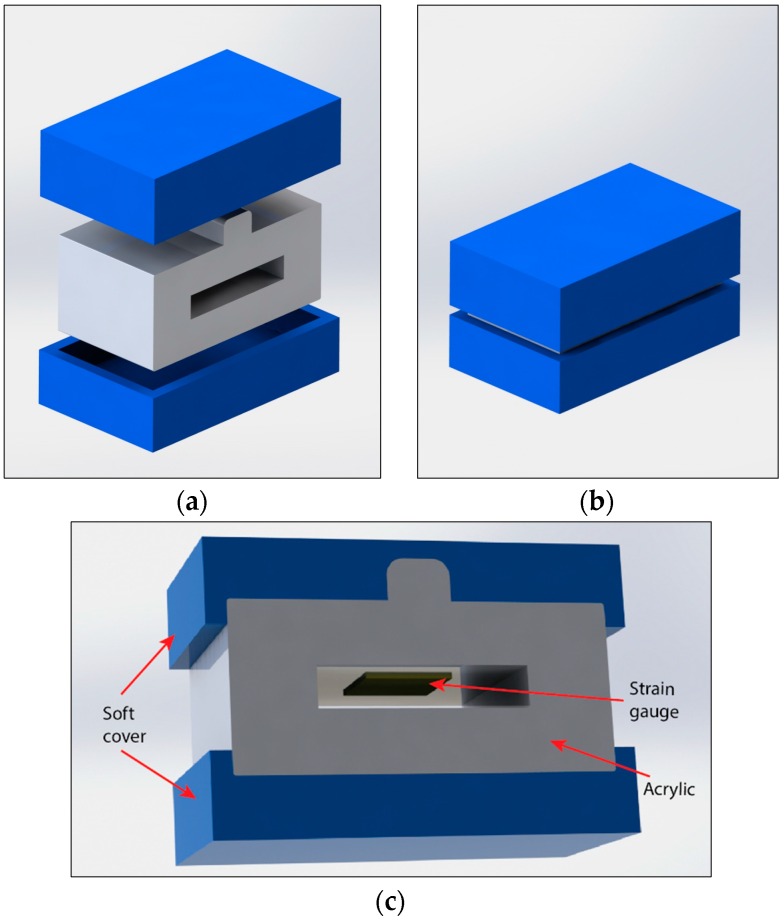
Sensor design. (**a**) Layered model; (**b**) Assembled sensor structure; (**c**) Cross-sectional view of the proposed sensor.

As mentioned in the introduction, the developed bite sensor should be capable of measuring forces ranging from 0 to 700 N. Additionally, the signal from the sensors should be stable and drift free to avoid measurement error. Furthermore, to simplify the conversion from output signal to an actual bite force, linear output properties are desirable for bite sensors. Based on these requirements, we proposed the design for the bite force sensor as shown in Figure 1. The sensor consists of two Poly-Vinyl Siloxane (PVS) addition silicone layers, an acrylic frame, and a metal strain gauge. Non-metal strain gauge devices such as fibre optic strain gauges [22] may provide more satisfactory results. However, these sensors are less common and do not satisfy the low-cost or ease of manufacturing we require. The PVS addition silicone functioned as a protective layer to reduce the potential pain caused to subjects when biting on the sensor. The PVS material used is a common impression material (Virtual Heavy Body Regular Set) used by dentists, with suitable properties for intraoral use [23,24]. The acrylic frame was used to transfer the mechanical strain, caused by biting force, to the strain gauge. We chose acrylic due to its high chemical inertness, low cost, ease of manufacturing, and worldwide availability. The strain gauge attached to the inner side of the acrylic and functions as the sensing element.

When a bite force is applied to the top and bottom of the sensor, it will deform the acrylic frame, inducing a strain on the strain gauge. Consequently, the gauge will change its resistance due to the modification of its dimensions. Therefore, by measuring the resistance change of the strain gauge, it is possible to estimate the applied bite force or MVBF.

**Figure 2:**
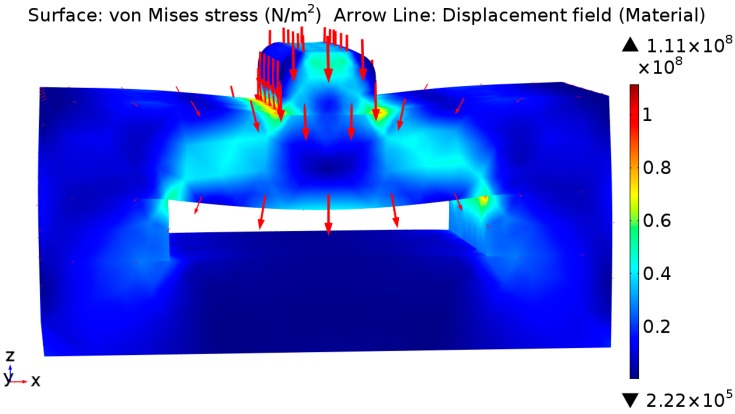
Stress distribution with bite force of 700 N.

Finite element analysis (FEA) simulations were performed using Comsol Multiphysics to investigate the strain induced in the sensor under an applied uniform force. Figure 2 shows the von Mises stress distribution on the sensor with a maximum stress of approximately 100 MPa under a maximum applied load of 700 N, which is lower than the strength of the acrylic material with a flexural strength of 116 MPa as per Perspex’s data sheet [25]. Simulation results also showed that a strain of approx. 1.2% would be applied to the strain gauge (load of 700 N).

## 3. Fabrication and Calibration

**Figure 3:**
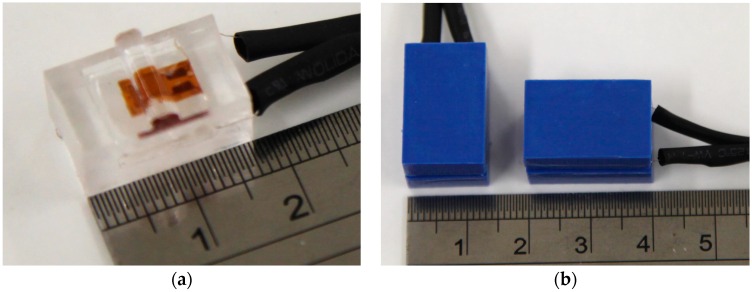
Fabricated sensor. (**a**) Prototyped sensor with bonded strain gauge; (**b**) Assembled sensor with protective silicone layer.

The designed sensor was fabricated using a Trotec Speedy 300 Laser Computer Numerical Control (CNC) machine to cut 10 mm thick Perspex cell cast acrylic sheet, with a resolution of 0.1 mm. This laser cutting method allows for mass production of the acrylic sensors. Once the cutting process was complete and the acrylic had been given enough time to cool down, the sensor was removed from the laser CNC machine. This process took approximately 5 min to complete. A strain gauge, with a Gauge Factor (GF) of approx. 2.1, was then inserted and attached to the inner-upper beam of the sensor. The manufacturing process of the sensor could be easily automated in order to produce larger quantities of sensing devices. Figure 3a shows a fabricated device with a bonded strain gauge.

In order to allow the patient to bite down on the sensor with less risk of harm or discomfort, we covered the sensor with a PVS addition silicone layer, as shown in Figure 3b. The protective layer was formed using a laser cut acrylic mould. The PVS addition silicone material was applied to the mould using a mixing gun and mixing tip [23]. Mould inserts were inserted into the mould and excess material was forced out of the mould, forming the protective layers. Excess material was cleaned off of the protective layers once removed from the mould.

**Figure 4:**
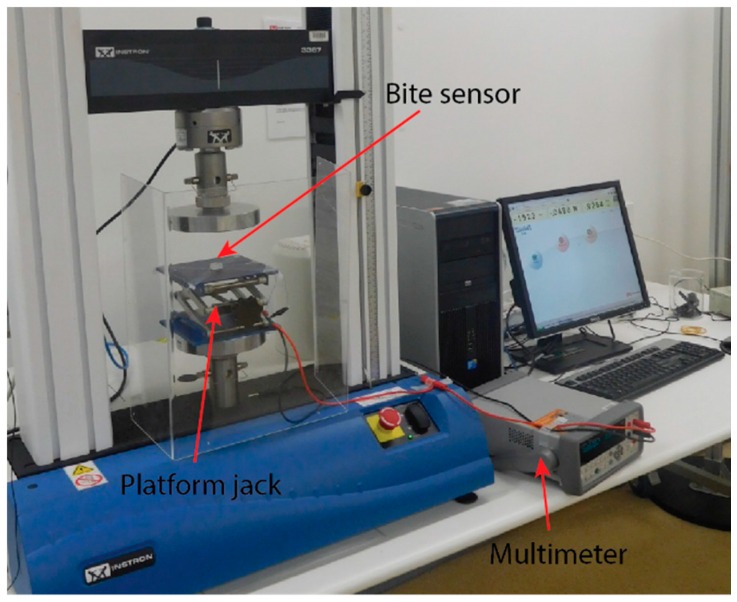
Calibration setup for compression tests.

The force was applied and measured externally using an Instron Model 3367 30 kN universal testing machine (referred to as an Instron machine) in order to take accurate and reliable measurements of the force exerted on the sensor. Figure 4 shows the experimental setup for the calibration of the bite force sensor.

Due to the nature of the high forces which are applied to the sample under compression using the base plate of the Instron machine, a platform screw jack was used to reduce the rate of increasing applied force by introducing mechanical deflection. With this deflection, we were able to measure with a higher precision and avoid unintentionally crushing the samples.

The sensor is positioned in the centre of the platform screw jack and positioned with maximum contact area on the Instron platform. The contact point of the sensor aligned with the centre of the upper plate of the Instron machine. Using the Instron’s manual controls, the platform was lowered down to the sensor, leaving a small gap to remain out of contact. In order to apply a load to the sensor, the Instron device is manually lowered slowly at a rate of 1 mm/min in increments of 50 and 100 N for static force measurements.

**Figure 5:**
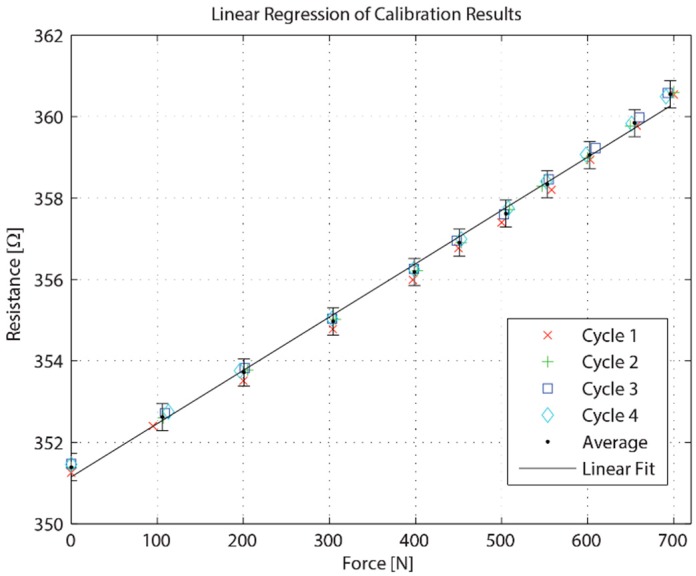
Calibration results.

The resistance change of the strain gauges in the bite sensors were measured using a high resolution multimeter (Agilent 34401), which can detect a relative resistance change of approximately 0.003%. This method allows for precision measurement; however, the recorded data is intermittent as seen in Figure 5.

A near-linear relationship between the applied force and measured resistance can be seen from the calibration results. Based on the simulation results, with 1.2% strain at 700 N, an expected resistance change of 2.52% was estimated for a strain gauge with a GF of 2.1. This estimation result agrees well with the experimental results shown in Figure 5. As such, at an applied force of 700 N, the resistance change is approximately 2.5%.

## 4. In Vivo Experimental Validation

To continuously record the resistance, we employed a Wheatstone bridge and a voltage amplifier (AD 623) connected to an oscilloscope (MSO-X 3104A, Agilent Technologies, Santa Clara, CA, USA). This circuit has been widely used in piezoresistive force sensors [26,27]. The output voltage of the bite sensor is proportional to the resistance change of the strain gauge as follows:

(1) $$V_{o}=\frac{V_{i}}{4}\frac{\Delta R}{R}K$$

where V_o_ and V_i_ are the output voltage and input voltage, respectively. K is the gain of the amplifier and $\frac{\Delta R}{R}$ is the relative resistance change of the strain gauge. The calibration results can then be used to convert the measured voltage into a readable force value [28,29].

**Figure 6:**
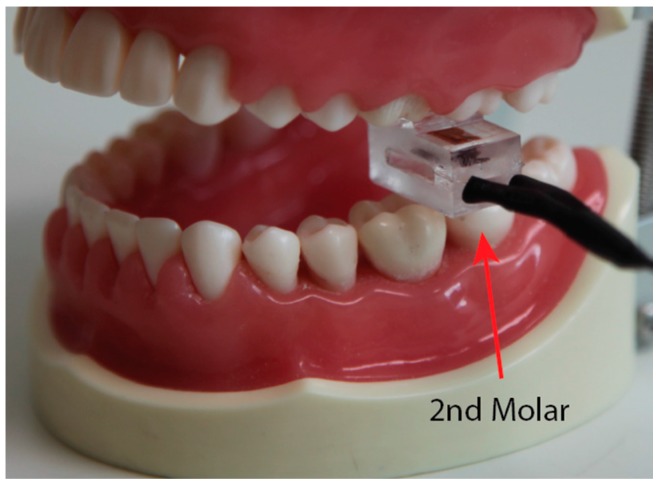
Position and orientation of sensor in practical testing.

A practical test was performed on three human subjects following GU Human Ethics Protocol 2016/142. The prepared sensor was disinfected and inserted into an air/water syringe cover for practical testing. A subject was asked to position the sensor under their second molar and make three consecutive attempts to bite as hard as they could. The tests were performed in a safe environment (dentist’s office) with the positioning method shown in Figure 6. 

**Figure 7:**
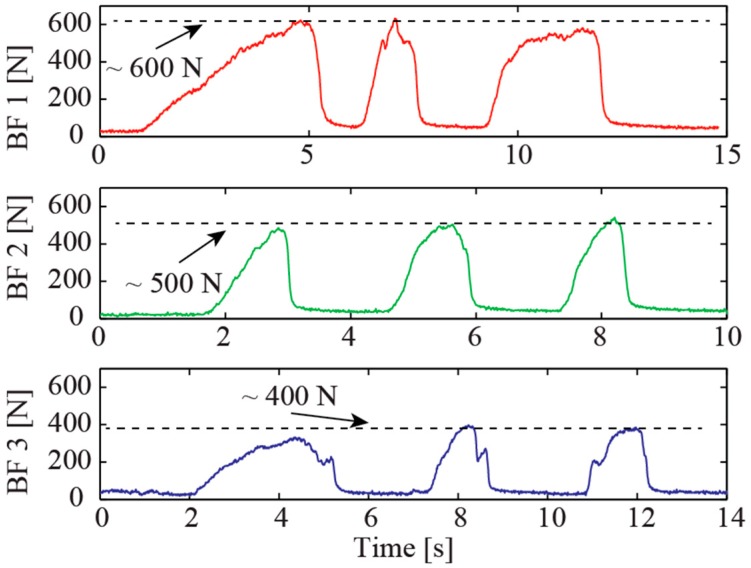
Bite forces of human subjects.

Two of the three experiments were performed on subjects with healthy teeth. The third subject had dentures. The bite forces noted were 500 N and 600 N in healthy male subjects and 400 N for the subject with dentures, as shown in Figure 7. These values all fell within the expected values of MVBF readings. It is worth noting that the responses of the sensor to human bite forces is with a high signal-to-noise ratio. This indicates that the sensor can be used for highly sensitive monitoring of oral status.

In addition, the gradual increase in force for the first bite of each subject was observed indicating a common tendency of the subjects to first get a feel for the bite force sensor before making further attempts. The results all exhibit evidence for a good performance of the novel bite force sensor which may find applications in oral status monitoring and other healthcare applications.

The evaluation in Table 1 has the following notes:

1. Fabrication (and structure)—simple: O (compact structure and easy to fabricate); complex: Δ (requires sophisticated processes, such as CNC machining); very complicated: X (time consuming process and bulky structure, such as hydraulic structures).
2. Cost—low: O (worldwide availability and simple fabrication); moderate: Δ (using commercially available sensors with non-complicated structures); high: X (complicated and time consuming processes, which may not be relevant to mass production).
3. Measureable range (approximation in some cases)—>600: O; 300–600: Δ; <300: X.

The above calibration and demonstration experiments have proven the potential of our strain gauge and acrylic based sensor for bite force measurement. This can be seen by the evaluation performed in Table 1. The first advantage of the proposed sensor lies in its cost. With the simple structure and worldwide availability of materials used in the sensor, the material cost of each sensor is estimated to be below 2 USD. In addition, with the possibility of mass production using the proposed laser cutting process, our developed sensor is expected to be extremely cost-effective. Since acrylic can be patterned using a laser cutter, which is less time-consuming than metal processing, our developed platform is also preferable for mass production. Despite acrylic being softer than metals such as steel and chromium, the use of a double side support design in our bite force sensor not only allows for the miniaturisation of the device size, but also offers a large measurable range of up to at least 700 N. Since the signal of the strain gauge can be directly converted to an output voltage using a Wheatstone bridge, the data processing of the developed device is easier than other force sensors utilizing piezoelectric and photo-diode based pressure transducers.

## 5. Conclusions 

In conclusion, the results presented herein demonstrate the simple and user-friendly fabrication of a novel, economical bite force sensor using an acrylic structure with a bonded metal strain gauge. The sensors showed good linearity and excellent repeatability with the capability of measuring a large bite force range of up to 700 N. The successful demonstration of the measurement of human bite force in real-time indicates good feasibility for using this novel sensor for personal healthcare applications.

## Figures and Tables

**Table 1:** Brief overview and evaluation of available bite force sensors. In order of appearance in main text. “-” depicts unknown or unsure.

Ref.	Sensing Principle	Sensor Material/Device Material	Max (N)	Mean (N)	Evaluation		
					Fabrication (i)	Cost (ii)	Measure Range (iii)
[1]	“specially designed transduction device”	-	392	344.9	-	-	Δ
[2]	Model 13 sub-miniature load cell	HSS, tool steel prongs	323.3	180.6	Δ	Δ	Δ
[4]	Pressure transducer (Omega PX300)	Pressurised rubber tube	1280	814	X	Δ	O
[5]	Strain gauge	316 stainless steel fork	-	615.8	Δ	Δ	Δ
[6]	Custom piezoresistive composite	Conductive carbon black powder and PDMS embedded in acrylic splints	120 range	-	Δ	Δ	X
[7]	Strain gauge	“high quality spring steel”	668	606.8	Δ	Δ	O
[8]	Model 13 sub-miniature load cell	316L stainless steel, shaped like mouth guard	146.7	101.01	Δ	Δ	X
[30]	Tekscan FlexiForce	Stainless steel plates	-	62.23	Δ	Δ	O
[31]	“expansion measurement strips”	Hardened tool steel with chromium plating fork	428.78	168.03	Δ	Δ	O
[32]	Digital dynamometer (Kratos DDK/M)	-	-	354.01	-	-	-
[33]	Hydraulic pressure gauge	“vinyl material encased in disposable plastic tube”	825.5	779	X	-	O
[34]	3-axis load cell (Kistler 9251A)	Acrylic plates	888	545.7	Δ	X	O
[35]	Pressure transducer (Omega PX309)	Water filled flexible synthetic tube with outer PVC tube and soft silicone tube coating	<1000	577	X	X	O
[35]	Strain gauge	T-shaped metal with EVA sheet covers	<400	254	Δ	Δ	Δ
[36]	Tekscan FlexiForce	Acrylic splints	-	619.8	O	Δ	O
[37]	Strain gauge (Occlusator)	Stainless steel	-	306.07	-	Δ	Δ
[38]	Digital dynamometer (Kratos IDDK)	-	999.3	590	-	Δ	O
[39]	Tekscan FlexiForce	Acrylic splints	-	249.8	O	Δ	O
This work	Strain gauge	Acrylic	700	-	O	O	O
